# Continuous noninvasive blood gas estimation in critically ill pediatric patients with respiratory failure

**DOI:** 10.1038/s41598-022-13583-6

**Published:** 2022-06-14

**Authors:** Junzi Dong, Minnan Xu-Wilson, Bryan R. Conroy, Robinder G. Khemani, Christopher J. L. Newth

**Affiliations:** 1 Connect Care and Personal Health, Philips Research North America, 222 Jacobs Street, Cambridge, MA 02141 USA; 2 Department of Anesthesiology and Critical Care Medicine, Children’s Hospital Los Angeles, Los Angeles, CA USA; 3 Department of Pediatrics, Keck School of Medicine, University of Southern California, Los Angeles, CA USA

**Keywords:** Paediatrics, Scientific data

## Abstract

Patients supported by mechanical ventilation require frequent invasive blood gas samples to monitor and adjust the level of support. We developed a transparent and novel blood gas estimation model to provide continuous monitoring of blood pH and arterial CO_2_ in between gaps of blood draws, using only readily available noninvasive data sources in ventilated patients. The model was trained on a derivation dataset (1,883 patients, 12,344 samples) from a tertiary pediatric intensive care center, and tested on a validation dataset (286 patients, 4030 samples) from the same center obtained at a later time. The model uses pairwise non-linear interactions between predictors and provides point-estimates of blood gas pH and arterial CO_2_ along with a range of prediction uncertainty. The model predicted within Clinical Laboratory Improvement Amendments of 1988 (CLIA) acceptable blood gas machine equivalent in 74% of pH samples and 80% of PCO_2_ samples. Prediction uncertainty from the model improved estimation accuracy by 15% by identifying and abstaining on a minority of high-uncertainty samples. The proposed model estimates blood gas pH and CO_2_ accurately in a large percentage of samples. The model’s abstention recommendation coupled with ranked display of top predictors for each estimation lends itself to real-time monitoring of gaps between blood draws, and the model may help users determine when a new blood draw is required and delay blood draws when not needed.

## Introduction

Patients in severe respiratory distress are often supported by intubation with mechanical ventilation. The correct level of ventilation is critical for life support without further lung injury. Blood gas pH and arterial CO_2_ pressure (PaCO_2_) obtained through invasive blood draws are relied upon to help determine ventilator settings. In the acute phase of injury, frequent blood draws are needed to determine blood gases^1^. This is especially difficult in pediatric patients where arterial access, pain, and blood loss are major concerns^2^; moreover, arterial catheters are an under-recognized source of infection^3^. Improvements in pulse oximetry providing continuous monitoring of oxygenation has proved helpful in children and shifted practice patterns in pediatric intensive care to reduce use of arterial catheters^1, 2^. With respect to ventilation, exhaled CO_2_ monitored through capnography is correlated with blood gas (BG) CO_2_ tension but has not been accepted to provide the accuracy continuous monitoring oximetry does. However, the frequency of BG sampling is decreased with capnography usage^4–6^, demonstrating that clinicians informally use capnography to determine the direction of blood pH changes.

There has long been interest in estimating BG pH and PCO_2_ from end-tidal CO_2_ (PetCO_2_)^7–9^ and over the past few years there have been some stimulating new investigations on estimating these in pediatric patients noninvasively^10–13^. These studies show that PetCO_2_ concentrations along with other noninvasive measurements can be used to estimate the values of blood pH and PCO_2_ without taking an invasive blood sample. Nonetheless, challenges to clinical adoption remain. Prediction accuracy outside the normal pH range is low^10, 11^, and there is a lack of clinical confidence in the predicted values.

The goal of this study is to develop continuous BG estimation that is accurate in all pH ranges for mechanically ventilated children with a wide range of severity of lung injury and hemodynamic support. Special consideration was given to develop a model suitable for clinical adoption. Estimations are made with a prediction uncertainty range, and the model can abstain from making inaccurate estimations when prediction uncertainty is high in case of large physiological fluctuations. Investigations on estimation accuracy over time provides guidance on the timeframe in which continuous noninvasive monitoring can be used. To further help users interpret and understand an estimated BG value, predictors are ranked by those with most significant contributions to the estimated value and displayed. We developed the model on a large derivation dataset spanning 5 years of data using novel modeling techniques and tested it on unseen validation data.

## Methods

Following the guidelines of the Transparent Reporting of a Multivariate Prediction Model for Individual Prognosis or Diagnosis^14^, we developed and validated a BG estimation model that either provides an estimate of the current pH and PCO_2_ or abstains from estimation.

### Study population

**Figure 1:**
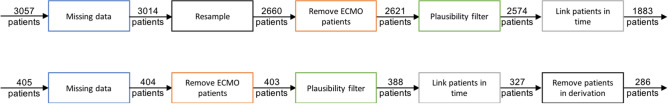
Block diagrams of derivation and validation cohort sizes and extraction steps.

The retrospective derivation and validation datasets were collected from pediatric critical care patients admitted to a tertiary pediatric intensive care center with a multidisciplinary pediatic medical–surgical ICU (PICU) and a pediatric cardiothoracic intensive care unit (CTICU), as shown in Table 1. Figure 1 illustrates data extraction steps for both cohorts. Derivation and validation cohorts spanned different times, and samples from the same patient could not appear in both cohorts. The dataset was approved with waiver of informed consent by the Children’s Hospital Los Angeles Institutional Review Board and the study protocol was approved by the Philips Internal Committee for Biomedical Experiments. All experiments were performaned in accordance with relevant guidelines and regulations.

**Table 1:** Cohort summary of final PICU and CTICU cohorts. IQR: interquartile range. AVDSf: alveolar dead-space fraction.

	PICU	CTICU
Subjects, n	902	1292
No. of observations	6681	9610
No. of observations per subject, median (IQR)	3 (1–9)	4 (2–9)
Time between BG (h)	5.1 (3.2–7.7)	4.3 (2.5–6.5)
Age, mo, median (IQR)	60.6 (16.4–151.1)	1.0 (0.0–7.2)
Weight, kg, median (IQR)	18.0 (9.6–39.4)	3.6 (2.9–6.4)
Female (%)	42.8%	42.2%
**Arterial blood gas, median (IQR)**		
pH	7.35 (7.30–7.43)	7.39 (7.34–7.46)
PaO_2_ (mmHg)	89 (69–116)	75 (45–121)
PaCO_2_ (mmHg)	45 (38–54)	44 (39–49)
**Noninvasive support**		
SpO_2_ (%)	98 (96–100)	97 (85–100)
PetCO_2_ (mmHg)	40 (34–47)	38 (33–43)
**Ventilator settings**		
Peak inspiratory pressure (cmH_2_O)	24 (19–30)	20 (17–24)
PEEP (cmH_2_O)	8.0 (5.3–10.0)	5.5 (5.0–7.0)
Mean airway pressure (cmH_2_O)	13.5 (10.0–17.2)	9.7 (8.0–11.5)
FiO2 (%)	40 (33–60)	40 (35–60)
Tidal volume (exp) (mL/kg)	7.2 (5.5–8.9)	7.5 (5.7–8.8)
Minute ventilation (L/min/kg)	153.8 (110.2–211.9)	203.7 (165.7–244.4)
**Lung disease severity**		
OSI	8.7 (5.3–13.2)	4.6 (3.0–6.9)
OI	5.7 (2.9–12.2)	4.8 (3.1–9.4)
SpO_2_/FiO_2_	238 (163–286)	228 (161–278)
PaO_2_/FiO_2_	211 (128–323)	186 (115–282)
AVDSf	0.11 (0.01–0.21)	0.13 (0.04–0.22)

#### Derivation cohort extaraction

The derivation dataset was collected from patient measurements made between September 2012 and May 2017 and stored prospectively in the hospital’s dedicated critical care SQL Server (Microsoft, Redmond, WA, USA). A dataset containing BG, granular physiological and ventilator data collected within ± 1 min of BG sample time was extracted from these medical records. pH and PCO_2_ measurements were obtained from both arterial and capillary blood gases, which made up 90% and 10% of the data samples, respectively. In model development and analyses, arterial and capillary BG were used inter-changeably given closeness of capillary BG to arterial BG^15^. Samples with missing information in PetCO_2_ measurement or medical record number (MRN) were removed. Derivation data was resampled to balance pH distribution and improve model performance in sparsely represented pH regions (eFig. 1). Patients on extracorporeal membrane oxygenation support were removed. A plausibility check was performed on measurement values as shown in eTable 1. Samples for the same patient were linked in time, and samples without a prior BG within 24 h were removed. Processing was done to ensure that variables were correctly linked in time, and that outcome variables (pH and PaCO_2_) were always linked to prediction variables measured prior in time. The final derivation cohort was split into 5 outer- and 5 inner-folds for cross-validation (CV) using nested CV^16^ stratified by pH. Predictor and model selection were performed on inner CV folds, and final training was done on all folds.

#### Validation cohort extraction

The validation dataset was collected from measurements made between June to December 2017. The pH resampling step was not done for the validation dataset, in order for validation performance to reflect a natural BG distribution. Patients in the validation cohort who had already appeared in the derivation cohort were removed.

### Predictors and target

Suppose the current time is $t$ and the previous BG was measured at time $t-1$. The target and predictor variables are shown in Table 2. ‘Delta’ predictors are the difference between current (taken at time $t$) and previous ($t-1$) measurements, e.g., $\Delta Sp{O}_{2}=Sp{O}_{2} [t]-Sp{O}_{2}[t-1]$. Final predictors included in the model were selected from 23 candidate predictors (eTable 2). Predictors were selected on inner CV folds using ridge regression to remove co-linearity between predictors and spurious correlations between predictors and targets.

**Table 2:** Targets for prediction, and predictors included in the final model.

Target variables $$pH\left[t\right]$$*,* $${PaCO}_{2}\left[t\right]$$
Predictor variables $$pH\left[t-1\right]$$, $${PaCO}_{2}[t-1]$$, $${{HCO}_{3}}^{-}\left[t-1\right]$$, $${etCO}_{2}\left[t\right]$$, $${etCO}_{2}\left[t-1\right]$$, $$\Delta {FiO}_{2}$$, $$\Delta PEEP$$, $$\Delta PIP$$, $$\Delta MnAwP$$, $$\Delta Sp{O}_{2}$$, $$\Delta TVin$$,$$\Delta TVexp$$

### Statistical analysis

#### Model building

A novel pairwise regression model was developed to model interactions between one key predictor (previous pH) and non-key predictors. This model allows differences in physiology between patients in different pH ranges to be modeled independently while representing monotonic relationships between non-key predictors and BG. The model is mathematically expressed as

$$\widehat{y}={\sum }_{j=1}^{M}{\sum }_{i=1}^{K}{w}_{i, j}\cdot {f}_{j}(z)\cdot {x}_{i},$$

where $\widehat{y}$ denotes the predicted target, ${x}_{i}, i=1,\dots , K$ denotes $K$ non-key predictors, $z$ denotes the key predictor, and ${f}_{j}\left(z\right), j=1,\dots ,M$ denotes sigmoid functions centered at $M$ different values of the key predictor.

**Figure 2:**
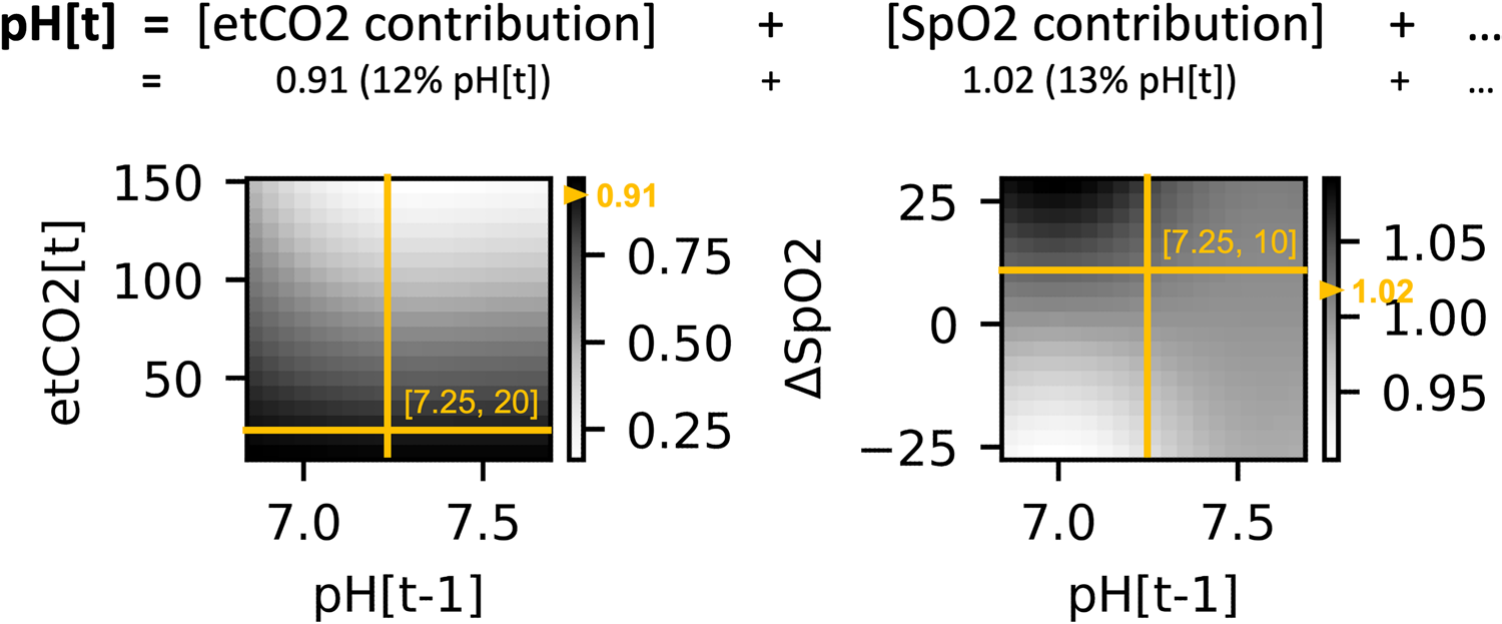
Examples of learned non-linear pairwise relationships between non-key predictors and the key predictor. The key predictor on the x-axes, previous pH (pH[t-1]), is shown with non-key predictors etCO_2_ and ΔSpO_2_ (left Y-axes). The predicted pH (pH[t]) is the sum of contribution from all predictors. Contribution of each non-key and key predictor pair to the total estimated pH is color-coded, with white indicating higher contributions and black indicating lower contributions (right Y-axes). A prediction example is shown for a hypothetical patient with previously measured pH of 7.25, current etCO_2_ of 20,and ΔSpO_2_ of 10 (denoted by the crossing points of the horizontal and vertical yellow bars). The predicted contribution for pH is read from the colormap, denoted by the yellow tick mark. The etCO_2_, ΔSpO_2_, and previous pH contributions are 0.91 (12% of predicted pH) and 1.02 (13% of predicted pH) from the learned relationships, respectively, and the total predicted pH is the sum of all contributions. The symbol ‘…’ denotes other predictor contributions not shown.

The model is interpretable: pairwise interactions can be visualized^17^ as show in Fig. 2 and the contribution of each predictor can be separated to generate predictor importance rankings for each estimation. Data processing, modeling, and analyses were performed in Python.

#### Prediction uncertainty around point-estimates and abstention

Prediction uncertainty was modeled to abstain from making predictions on high uncertainty samples and only accept predictions likely to be more accurate. Abstention rate was empirically set to 25%, meaning that 75% of samples were estimated.

Prediction uncertainty was modeled using bootstrap estimation of uncertainty^18^, by building separate models for each derivation CV fold and quantifying the agreement between them. For a given sample, the prediction uncertainty is the variance in predicted target values by all separate models. Only samples with uncertainty lower than a threshold generated an estimation, and this threshold was determined with the pre-defined criteria of improving the 95% percentile of pH predictions to ± 0.1 pH unit or lower while not abstaining on more than half of patients. Estimations were abstained on samples with high uncertainty.

The point-estimate of BG is generated from a final model trained on all derivation data. Separate models from derivation CV folds provide a prediction uncertainty range around the point-estimate.

#### Predictor importance ranking

When an estimate is made, predictor contribution to the estimation is ranked. The model can be rewritten as $\widehat{y}={\sum }_{i=0}^{K}g({x}_{i})$, which denotes the sum of contributions from individual predictors $g\left({x}_{i}\right)={w}_{i, j}\cdot {f}_{j}(z){\cdot x}_{i}$.

Given a sample $$\overset{\lower0.5em\hbox{$\smash{\scriptscriptstyle\rightharpoonup}$}}{x} =[{x}_{0}, {x}_{1}, \dots ,{x}_{i}, \dots , {x}_{K}]$$, the importance of the $$i$$ th predictor is

$${I}_{i}= {f}_{i}\left({x}_{i}\right)- {f}_{i}\left({\overline{x} }_{i}\right),$$

where ${\overline{x} }_{i}$ denotes the population mean of the predictor. When ${x}_{i}$ is close to ${\overline{x} }_{i}$, ${I}_{i}$ will be equal or close to 0, which means predictor ${x}_{i}$ contributes little to the overall estimate. When ${x}_{i}$ deviates from the population mean, ${I}_{i}$ shifts away from 0 to highlight the increased contribution of ${x}_{i}$.

#### Baseline models

The alveolar dead-space fraction (AVDSf) model was used to establish a baseline for comparison. It uses the alveolar dead-space fraction ($AVDSf= (PaC{O}_{2}-PetC{O}_{2})/PaC{O}_{2}$) calculated from the previous BG to estimate the current PCO_2_. This estimate for PCO_2_ is used along with the previous HCO_3_^−^ to estimate the current pH using the Henderson-Hasselbalch equation. A capnography-free linear regression model by Baudin et al.^10^ using PetCO_2_, FiO_2_, and mean airway pressure (MnAwP) was also tested on the validation dataset for comparison.

#### Performance comparison

Performance was evaluated by the 95% percentile of absolute error, or the worst 5% of samples. Performance of samples in separate pH ranges was reported. The percentage of samples with absolute error under 0.04 pH unit or 5 mmHg PCO_2_ was calculated, following the CLIA gold standard for blood gas^19^.

## Results

### Cohort characteristics

Characteristics of the final derivation and validation cohorts are described in Table 3. The cohorts are representative of a general pediatric intensive care population. Sub-cohort criteria such as pediatric acute respiratory distress syndrome (PARDS) and respiratory acidosis are defined in Supplementary material.

**Table 3:** Summary of derivation and validation datasets.

	Derivation dataset	Validation dataset
Final # of patients	1883	286
Final # of BG samples	12,344	4030
Age, mo, mean ± STD	50 ± 73	40 ± 62
CTICU	60% of patients	57% of patients
PICU*	41% of patients^†^	43% of patients
Metabolic acidosis^‡^	14%	10%
Respiratory acidosis^‡^	28%	22%
Metabolic alkalosis^‡^	7%	17%
Respiratory alkalosis^‡^	6%	10%
Mixed^‡^	44%	41%
PARDS^‡^	**12%**	**9%**
Before resampling	**11%** pH < 7.3	**7%** pH ≤ 7.3
	**71%** 7.3 ≤ pH < 7.45	**71%** 7.3 ≤ pH < 7.45
	**18%** pH ≥ 7.45	**22%** pH ≥ 7.45
Post resampling and processing	**17%** pH < 7.3	NA^§^
	**54%** 7.3 ≤ pH < 7.45	
	**29%** pH ≥ 7.45	
Blood gas type	**90%** arterial	
	**10%** capillary	

Numbers for post-processed data are shown, except for pH range data. *STD* standard deviation. *PICU pediatric (multidisciplinary, medical-surgical) ICU. †Patients may have stayed in both ICUs. ^‡^Definition of PARDS, respiratory and metabolic acidosis and alkalosis are discussed in Supplementary material. ^§^Validation dataset was not resampled.

### Validation performance

**Figure 3:**
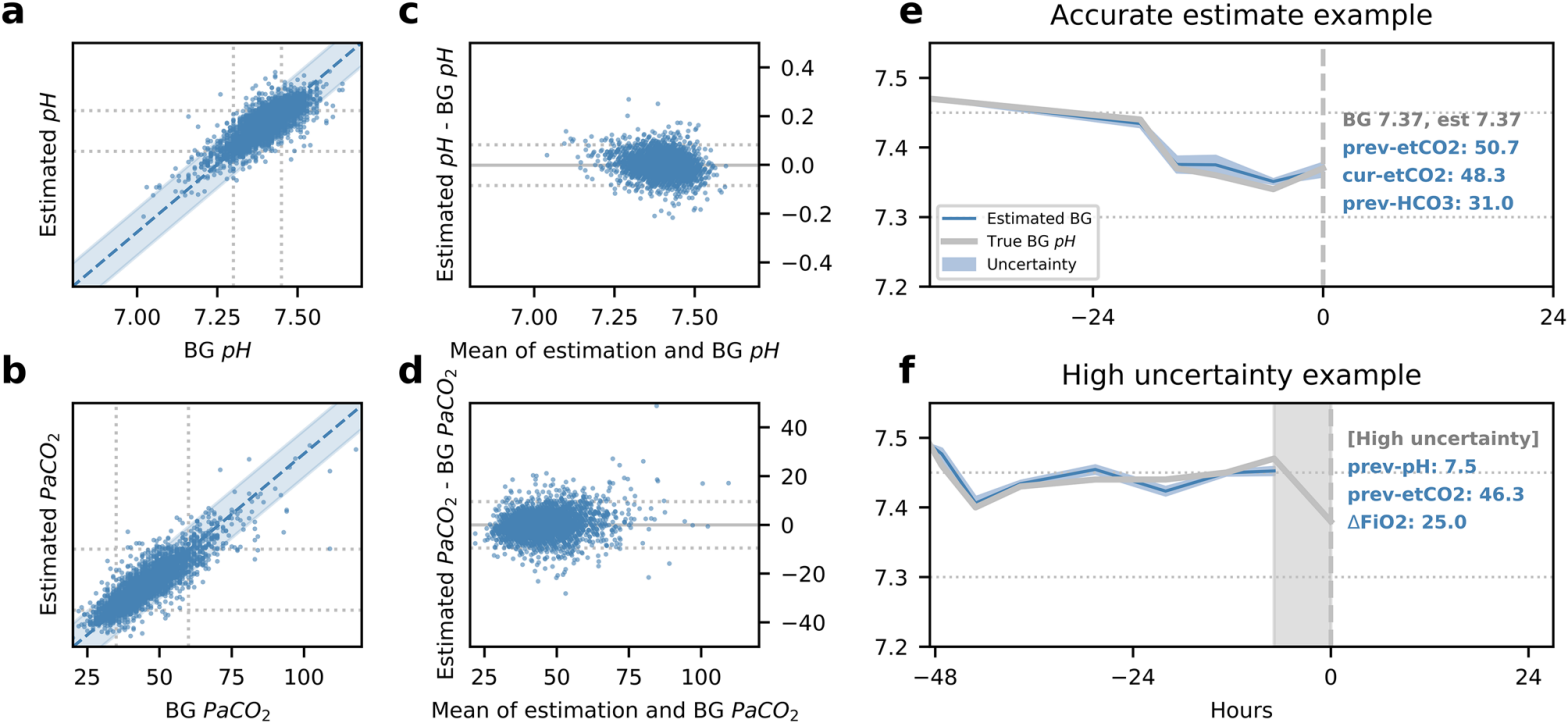
Predicted blood gas (BG) pH and PCO_2_ results on validation samples. Subplots (**a**,**b**) show the scatter plots of the model generated point-estimate and laboratory-derived pH and PCO_2_, while (**c**,**d**) show Bland–Altman plots for these estimations. Subplot (**e**) shows a patient example where the estimate at time $\mathrm{t}=0$ is made accurately with low uncertainty, and (**f**) shows a patient example where the estimate at time $\mathrm{t}=0$ is abstained on the basis of high prediction uncertainty. In the scatter plots (**a**,**b**), the blue shaded regions are the 95% percentile for all samples. The three pH or PCO_2_ regions are separated by vertical and horizontal dashed lines. In the Bland–Altman plots, the middle solid line shows mean predicted error, and the top and bottom dashed lines show ± 1.96 standard deviation.

Figure 3 plots estimates of pH and PCO_2_ against laboratory values for the validation dataset, and estimation performance before and after abstention are shown in Table 4. Overall, estimations were within CLIA acceptable blood gas machine equivalents^19^ in 74% of pH samples (± 0.04 pH unit) and 80% of PCO_2_ samples (± 5 mmHg). Estimation accuracy was balanced across pH, especially after abstention. Using the Mann–Whitney U-test, the validation results outperformed AVDSf and Baudin^10^ models (eTable 4) with statistical significance P value < 0.001.

**Table 4:** Blood gas estimation performance on derivation and validation datasets before and after abstention.

	Derivation		Validation	
	Before abstention	After abstention	Before abstention	After abstention
**pH 95% percentile (± pH units)**				
All	0.103	0.092	0.086	0.078
< 7.3	0.114	0.096	0.102	0.083
7.3–7.45	0.103	0.094	0.081	0.075
≥ 7.45	0.089	0.076	0.096	0.083
**PCO**_**2**_** 95% percentile (± mmHg)**				
All	10.33	8.78	9.67	8.72
20–35	9.65	7.82	9.33	7.82
35–60	9.45	8.43	9.08	8.52
60–120	17.43	13.78	17.95	13.48

### Prediction uncertainty and predictor importance

**Figure 4:**
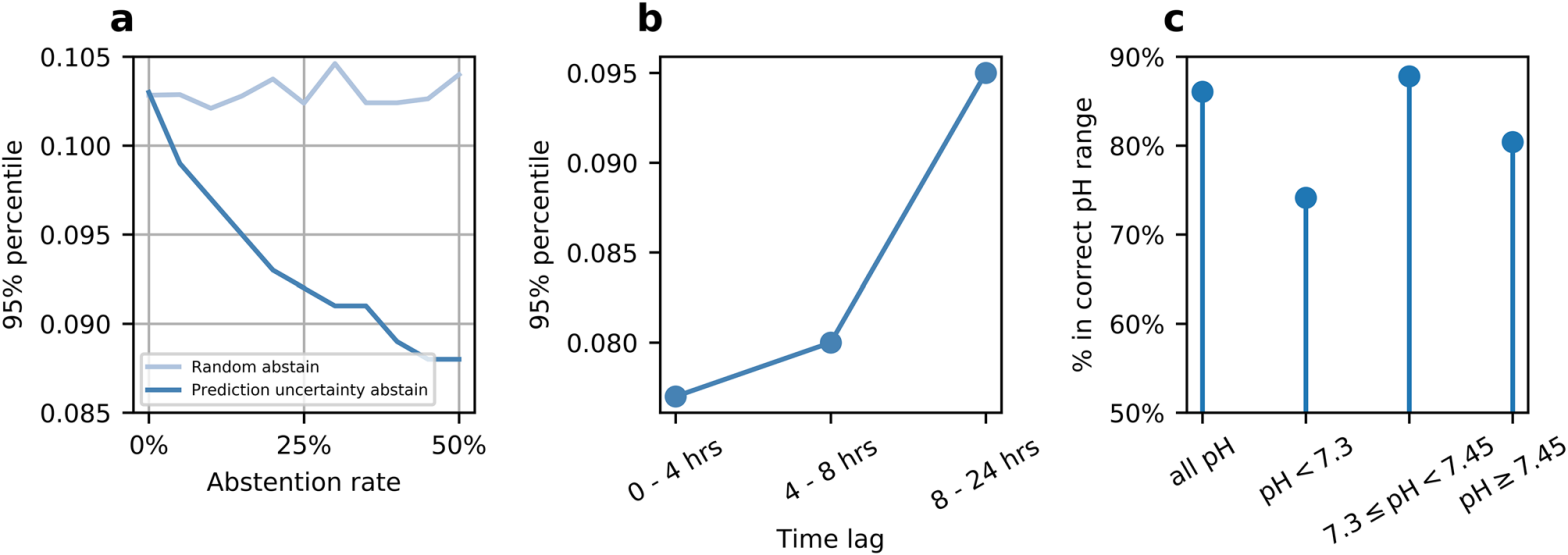
(**a**) Demonstrates that using prediction uncertainty to abstain on high-uncertainty samples improves estimation accuracy, while randomly abstaining the same percentage of samples provides no accuracy improvement. (**b**) Compares the estimation performance between samples with different time lags, defined as the time passed since the last BG. (**c**) The percentage of estimations that fall in the correct range of pH after abstention.

Estimations were not made when prediction uncertainty was above an acceptable threshold, as shown in the example in Fig. 3f. Also shown are the top three predictors ranked by importance and their measured values. The uncertainty threshold for abstention was obtained by examining the trade-off between performance and abstention rate on derivation samples, as shown in Fig. 4a. Abstaining using prediction uncertainty outperforms randomly abstaining the same percentage of samples, as shown in Fig. 4a, indicating that prediction uncertainty is a useful measure of estimation confidence.

### Prediction accuracy over time

Figure 4b examines the relationship between estimation accuracy and the time elapsed since the last BG. Samples were split into bins based on time lags. The 95% percentile remained under ± 0.080 until up to an 8-h time lag between the time of estimation and the previous BG.

While the most recent BG was used for modeling, an additional analysis examined using the first available BG for each patient for all subsequent estimations. The 95% percentile using the first available BG was ± 0.124 pH unit, compared to ± 0.078 pH unit when using the previous BG.

### Safe classification of pH

As the predicted values are used to guide ventilator settings, erroneous predictions between pH ranges could be potentially dangerous for patients. Figure 4c examines whether the estimated pH range, spanned by the point estimate plus uncertainty range, cover the correct pH range. Overall, 85% of all estimations cover the correct pH range, while those in individual pH-ranges are above 70%.

### Arterial and capillary blood gas

Estimations based on arterial BG were slightly more accurate than estimations based on capillary BG but not statistically different. The null hypothesis of no statistically significant difference in absolute estimation errors was not rejected by a Mann–Whitney U-test with P value 0.07.

### Model visualization

Figure 2 depicts two example non-linear relationships learned between non-key predictors and the key predictor, previous pH. Contribution to estimated pH is color-coded onto the two-dimensional predictor value space, with black indicating higher estimated pH contributions and white indicating lower estimated pH. The left plot shows that a lower etCO_2_ measurement contributes to a higher estimate of pH contribution, as seen in the dark color at the crosspoint where a low etCO_2_ measurement of 20 falls. If the same patient had a higher etCO_2_ measurement, the predicted pH contribution would be lower due to the crosspoint falling higher on the vertical line and into the lighter lower pH contribution zone. The right plot shows that the non-linear relationship between ΔSpO_2_ and pH also varies depending on the key-predictor, previous pH.

## Discussion

This study demonstrates that noninvasive parameters routinely available on most clinical monitors and ventilators can be used to provide useful estimates of BG in all intubated patients without necessitating a new blood draw, for up to 8 h. The model outperformed previous models while providing prediction uncertainty and predictor importance ranking, both of which can help users assess whether the model is likely to be accurate in a specific patient scenario. Built-in transparency of the model enables interpretation of estimation results, encouraging trust in adopting novel data-driven solutions for clinical practice.

The model estimated within CLIA acceptable blood gas machine equivalents^19^ in 74% of pH samples (± 0.04 pH unit) and 80% of PCO_2_ samples (± 5 mmHg). The model achieved better performance than previously reported models^10, 11^, especially in low-pH samples. Prediction accuracy on validation data was comparable to that on derivation data, demonstrating that the model is generalizable to new data.

The pH ranges in this study were used by the ARDSNet studies^20^. While accuracy in the absolute value of a pH estimation is important, users may be more concerned with whether pH is estimated in the correct range. For example, it would be very detrimental for a pH of 7.15 (low) to be estimated as 7.45 (high) since the likely change in ventilator management would be rather different under the two clinical situations, whereas an inaccurate estimation of 7.25 (compared to 7.15) would result in a less impactful modification to treatment and the change suggested would be in the same direction as that for the lower pH of 7.15. We showed that the majority of estimated samples cover the correct pH range in Fig. 4c. Low-pH samples remain the most challenging samples to estimate but using prediction uncertainty results in 74% of low-pH samples falling in the correct range.

Using older BGs for prediction was less accurate than using more recent BGs. This is likely due in part to variable changes in patient condition with time. Estimation accuracy decreased with longer time intervals between time of estimation and prior blood sample, but estimations remained accurate until up to 8 h, suggesting that typically one may abstain from blood draws up to 8 h from the previous BG.

The model utilizes readily available data sources in ventilated patients to provide continuous monitoring of BG through estimation. Estimations are made with prediction uncertainty, which highlight inherent uncertainty in the model and prevent the display of potentially inaccurate predictions. Furthermore, the model displays top predictors and their values, which gives the user more context around the estimation.

One could argue that in current practice, clinicians are already able to ‘guesstimate’ the BG pH based on the same data, and that any estimation that does not achieve laboratory-level performance is not useful. We propose there are merits of the model even at the current performance level. First, the model provides automatic and continuous monitoring of BG pH without any human effort, saving time and mental calculation even if the estimation is not perceived as better than a ‘guesstimation’. Second, ranked top predictors can illuminate patient measurements and changes that may not have occurred to bedside caregivers. Third, it can be a good reassurance model for clinicians who want to check that their ‘guesstimate’ matches trends from thousands of prior blood gases from which the model was developed. Finally, estimation uncertainty is displayed, and clinicians can always make sure that the model does no harm by opting to obtain a BG.

There are several potential applications of the model. First, noninvasive estimates of pH can decrease the number of blood draws further, and recommend that users obtain blood draws when they are most necessary. Second, continuously available estimates may facilitate standardized assessment of ventilator support and adherence to ventilator protocols, particularly those promoting lung protective recommendations. This has been implemented in the management of PARDS at Children’s Hospital Los Angeles^21,24^. Clinicians can accept or reject the protocol’s recommendation or obtain a blood draw if not confident about the prediction. In addition, the majority of BG for ventilated children in respiratory failure with PARDS lie in a normal to high range where the model performs well. Finally, the model has potential applications for closed loop ventilation, and will likely improve existing algorithms which use the PetCO_2_ directly.

The model uses a recent BG under the assumption that the patient’s respiratory and metabolic conditions have not drastically changed. Many external and contextual, and patient conditions are not available or captured at the time of estimation, so the final decision at the bedside must be left to the expertise of clinicians. One potential direction for improvement is obtaining a large dataset of BG, physiological measurements, and ventilation parameters along with full volumetric capnography for all patients. Volumetric capnography may provide additional information about patients’ respiratory states and prognoses^22, 23^ not present in PetCO_2_. which could enable more accurate estimation of BG. Lastly, although the model was generalized to unseen patients from the same center, it is unknown whether the model will generalize to other centers. This also requires validation on additional data.

The main application of the model is likely to be when ventilated patients have been stabilized and are in a relatively steady clinical state. Hopefully, it will be a viable tool for avoiding blood draws and facilitating continuous BG monitoring leading to more lung protective practices as we currently understand ventilation and oxygenation management^24^. Ventilator decision support protocols based on measurements of arterial BG have proven useful in the management of adult respiratory failure^20^. Accurate noninvasive measurements of arterial or capillary PCO_2_ with subsequent prediction of pH could allow more frequent ventilator changes to optimize lung and diaphragm protective ventilation without BG analysis, which would be particularly useful in pediatric practice where fewer arterial lines are used^25^.

The current model and results have some limitations. Specifically, model estimation only works on patients who have at least one recent blood gas invasively sampled. Abstention when model uncertainty is high defaults to invasive sampling and limits cases when noninvasive estimation can be used. Currently, there is no pre-determinant of which patients are likely to generate high uncertainty samples. All of these questions may be better answered in a randomized control trial, which would also provide information on the usefulness and safety of such a model at the bedside.

## Supplementary Information

Supplementary Information.

## Data Availability

The datasets analysed during the current study are not publicly available due to restrictions in IRB approved usage.

## Abbreviations

ABPd: Diastolic arterial blood pressure
ABPm: Mean arterial blood pressure
ABPs: Systolic arterial blood pressure
AVDSf: Alveolar dead-space fraction
BG: Blood gas
CLIA: Clinical Laboratory Improvement Amendments of 1988^19^
CTICU: Cardiothoracic intensive care unit
PICU: Pediatric (multidisciplinary, medical-surgical) intensive care unit
CV: Cross-validation
FiO_2_: Fraction of inspired oxygen
HR: Heart rate
IQR: Interquartile range
NBPd: Diastolic noninvasive blood pressure
NBPm: Mean noninvasive blood pressure
NBPs: Systolic noninvasive blood pressure
MnAwP: Mean airway pressure
OSI: Oxygen saturation index = $100\times {\mathrm{FiO}}_{2}\times MAP/{\mathrm{SpO}}_{2}$
OI: Oxygen index = $100\times {\mathrm{FiO}}_{2}\times MAP/{\mathrm{PaO}}_{2}$
PCO_2_: Arterial or capillary CO_2_ pressure
PaCO_2_: Arterial CO_2_ pressure
PcCO_2_: Capillary CO_2_ pressure
PARDS: Pediatric acute respiratory distress syndrome
PetCO_2_: Exhaled end-tidal CO_2_
PEEP: Positive end expiratory pressure
PIP: Peak inspiratory pressure
PFratio: PaO_2_/FiO_2_
SpO_2_: Pulse oximetry saturation
SFratio: SpO_2_/FiO_2_
TVexp: Expiratory tidal volume
TVin: Inspiratory tidal volume
% leak: % Gas leak around endotracheal tube during respiratory cycle
