# Supplementary material for: Continuous noninvasive blood gas estimation in critically ill pediatric patients with respiratory failure
Source: Sci Rep. 2022 Jun 14;12:9853. doi: 10.1038/s41598-022-13583-6 (PMC9198060; doi:10.1038/s41598-022-13583-6)
Supplement: Supplementary file 1 — Supplementary Information. [file 41598_2022_13583_MOESM1_ESM.docx]

**Supplementary Material**

**Continuous Noninvasive Blood Gas Estimation in Critically Ill Pediatric Patients with Respiratory Failure**

Junzi Dong PhD^1^, Minnan Xu-Wilson PhD^1^, Bryan R. Conroy PhD^1^, Robinder G. Khemani MD^2,3^, Christopher J.L. Newth MD, FRCPC^2,3^

**Affiliations:** ^1^Connect Care and Personal Health Team, Philips Research North America, Cambridge, MA; ^2^Department of Anesthesiology and Critical Care Medicine, Children’s Hospital Los Angeles, Los Angeles, CA; ^3^Department of Pediatrics, Keck School of Medicine, University of Southern California, Los Angeles, CA.

## **Contents**

**eMethods**

- Plausibility filtering for predictors
- Predictor candidates
- Resampling to balance pH distribution
- Patient sub-group analyses

**eResults**

- Detailed results of baseline models
- Performance on patient sub-groups

## **eMethods**

**Plausibility filtering for predictors**

As shown in the data extraction block diagram in Fig. 1, plausibility filters were applied to all extracted predictors to ensure erroneous values were removed. The plausibility ranges for each predictor are shown in eTable 1.

| **Bound** | **PetCO_2_** | **HCO_3_^-^** | **PIP** | **pH** | **SpO_2_** | **OSI*** | **FiO_2_** | **ABPd** | **PEEP** | **SF*** |
| --- | --- | --- | --- | --- | --- | --- | --- | --- | --- | --- |
| **Lower** | 15 | 10 | 8 | 6.75 | 70 | 0 | 0.21 | 10 | 0 | 100 |
| **Upper** | 145 | 50 | 45 | 7.65 | 100 | 45 | 1 | 120 | 25 | 425 |

**eTable 1** Plausibility range of measurements used to perform plausibility filtering during data extraction. *Only SpO_2_ values of 97 or less were used to calculate OSI and SF ratio.^E1^

**Predictor candidates**

The final predictors were selected from the full list shown in eTable 2. Selected predictors are shown in bold.

| ABPd | **FiO_2_** | **MnAwP** | **OSI** | **PIP** | **TV expired** |
| --- | --- | --- | --- | --- | --- |
| ABPm | Height | NBPd | **PetCO_2_** | **SpO_2_** | Weight |
| ABPs | HR | NBPm | **PEEP** | **SF ratio** | % Leak |
| Age (months) | **MinVol*** | NBPs | PICU or CTICU | **TV inspired** |  |

**eTable 2** Summary of all input measurements considered. Input measurements in bold were used in the models.*Minute ventilation

**Resampling to balance pH distribution**

The natural distribution of pH is concentrated in the normal physiological range, while extreme values of low- and high-pH are under-represented. This is part of the reason previous studies have reported poor performance in these pH ranges. To learn an optimal model for pH estimation, it’s important to have sufficient relative representation of samples in extreme pH regions and around the borders of normal to extreme values. To this end, re-sampling was designed to remove samples in the middle of the normal pH region away from borders with extreme values. eFigure 1 demonstrates the re-sampling strategy. Samples from under-represented regions were sampled with a resampling probability of 1, meaning that all low- and high-pH samples were kept. Meanwhile, samples from the over-represented region were resampled with pre-specified probability as indicated by the gray dotted line in eFig. 1b. Mid-pH samples furthest away from pH range borders were re-sampled with the lowest probability, while those closer to the borders were more likely to be re-sampled. This method generated a dataset that was more balanced across pH for model building, as shown in eFig. 1 and Table 2.

**eFigure 1** The resampling technique used to achieve a more balanced dataset in pH. **a)** Histogram of the original dataset shows that 73% of samples are between normal pH range, 7.3 and 7.45. **b)** Each pH region was sampled using the specified resampling probability. Low- and high-pH samples were resampled with a probability of 1, while samples in the mid-pH region were resampled with pre-defined probability under 1.

**Patient sub-group analyses**

Four binary (Y/N) patient sub-groups were created, and each patient was assigned four independent membership labels indicating which binary category she/he belonged to in each sub-group. The grouping criteria were: (1) age over or under 24 months, (2) patient has pediatric acute respiratory distress syndrome (PARDS) or not, (3) patient has pure or mixed metabolic or respiratory acid imbalance, and (4) patient’s ICU location (PICU or CTICU). The criteria for PARDS were defined as shown in eTable 3, and the criteria for metabolic or respiratory acid imbalance were defined as in Fig. 31-10 of the Guyton and Hall textbook^E2^. The criteria were applied to the first sample in each encounter, and then used to label all subsequent samples in the same encounter. Thus, a patient might be assigned to the patient group under 24 months, the group with PARDS, the group in PICU, and the group with pure metabolic or respiratory acidosis. Absolute estimation errors calculated by subtracting the point-estimate with the BG measurement and taking the absolute value were compared. Mann-Whitney U-tests were performed for absolute estimation errors for each sub-group category.

| **Criteria** | - PF O2 ratio ≤ 300, or - SF O2 ratio ≤ 264   OR   - OI ≥ 4, or - OSI ≥ 5   AND   - 2 serial samples fulfilling above criteria >= 4 hours apart   AND   - PICU |
| --- | --- |
| **PARDS prevalence** | 13.6% |

**eTable 3** Criteria for PARDS.

**Pairwise regression model formulation**

The model is mathematically expressed as

$\hat{y}=\sum_{j=1}^{M} \sum_{i=1}^{K} w_{i, j}\cdot f_{j}(z)\cdot x_{i}$*,*

where $\hat{y}$ denotes the predicted target, $x_{i}, i=1,\ldots, K$ denotes $K$ non-key predictors, and $z$ denotes the key predictor. It’s basically a combination of $M$ different linear regression models built at $M$ different key predictor (previous pH) values. During model building, the key predictor is split into $M$ bins, and a sigmoid $f_{j}\left( \right), j=1,\ldots,M$ centered at each $z_{j}$ is used to combine the $M$ models.

The model is interpretable: pairwise interactions can be easily visualized^17^ as show in Fig. 2 (further explained in Results), and the contribution of each predictor can be separated to generate predictor importance rankings for each estimation. Data processing, modeling, and analyses were performed in Python.

**Pairwise relationships learned by model**

**eFigure 3** All pairwise risks learned by the model.

## **eResults**

**Detailed results for baseline models**

| **pH 95% percentile (± pH units)** | **AVDSf** | **Baudin et. al** |
| --- | --- | --- |
| all | 0.115 | 0.128 |
| <7.3 | 0.136 | 0.138 |
| 7.3 – 7.45 | 0.090 | 0.105 |
| ≥7.45 | 0.117 | 0.122 |
| **PaCO2 95% percentile (± mmHg)** |  |  |
| all | 11.89 | 11.68 |
| 20 – 35 | 9.07 | 11.70 |
| 35 – 60 | 9.83 | 10.89 |
| 60 – 120 | 21.59 | 19.36 |

**eTable 4** Performance of the two baseline models for comparison.

The baseline models were tested on the validation dataset, and the results are shown in eTable 4.

**Patient sub-group analyses**

No patient sub-group categories were found to have consistent and statistically significant performance differences on both derivation and validation datasets.

## **eReferences**

E1.	Khemani, R. G., Patel, N. R., Bart III, R. D., & Newth, C. J. (2009). Comparison of the pulse oximetric saturation/fraction of inspired oxygen ratio and the PaO2/fraction of inspired oxygen ratio in children. Chest, 135(3), 662-668.

E2.	Hall JE. Guyton and Hall Textbook of Medical Physiology. Thirteenth. Elsevier Health Sciences; 2016.
